# Supplementary material for: Functional specialisation of multisensory temporal integration in the mouse superior colliculus
Source: Nat Commun. 2025 Oct 30;16:9615. doi: 10.1038/s41467-025-64600-x (PMC12575753; doi:10.1038/s41467-025-64600-x)
Supplement: Supplementary file 1 — Supplementary Information [file 41467_2025_64600_MOESM1_ESM.pdf]

Supplementary Figures

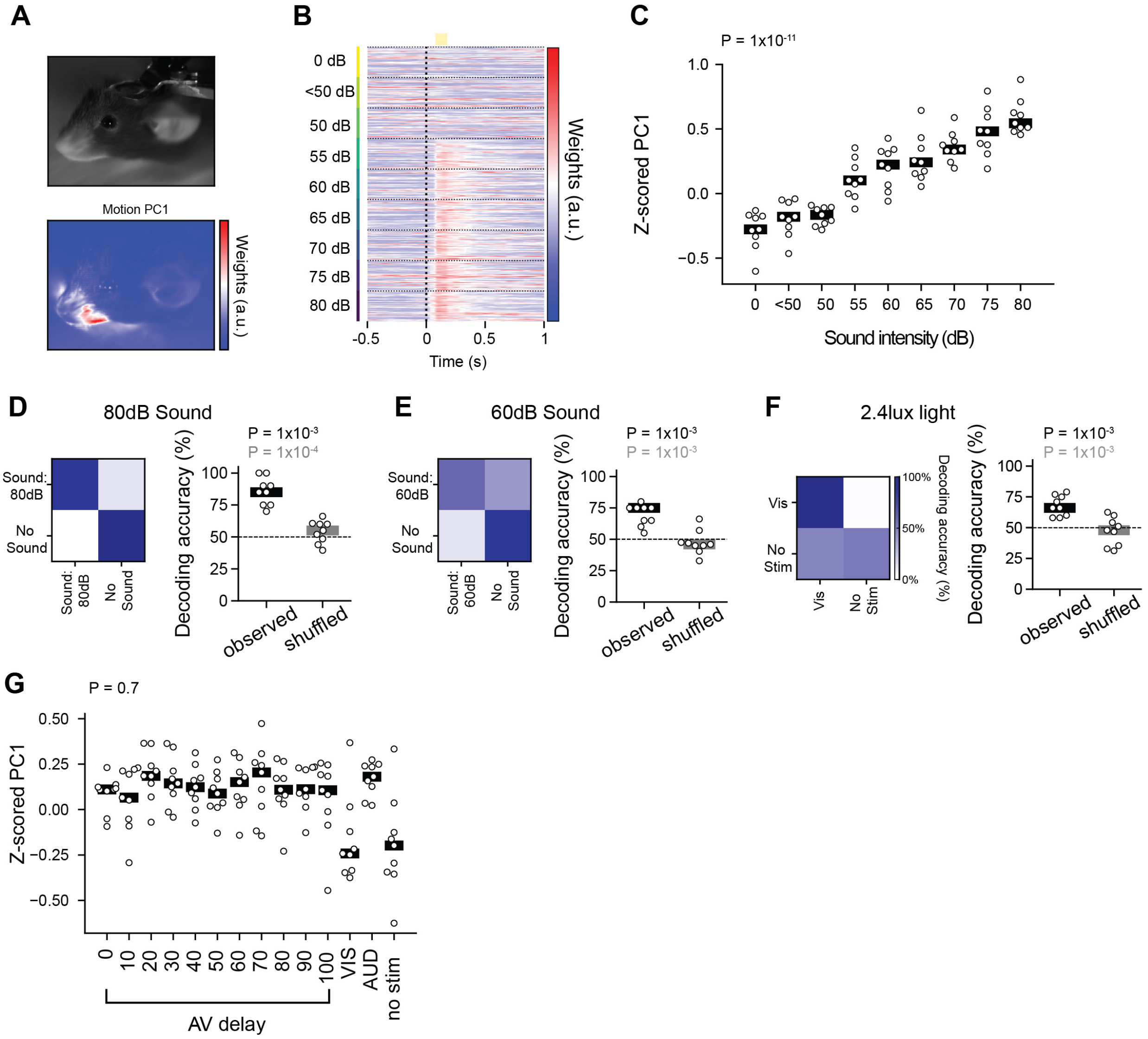

Supplementary Figure 1. Decoding sensory stimuli and audiovisual timing from motion energy representations.

A. Example video frame and spatial mask of the first principal component (PC) of the motion energy. Colour scale indicates low- (blue) to high-motion energy (red). **B**. Example representation of motion energy weights for the first PC over time. Data are aligned to stimulus onset (dashed line) and categorised by sound intensity (dB). **C**. Average z-scored motion energy weights for the first PC for the different sound intensity trials. P value from Kruskal-Wallis test. Individual datapoints represent individual recordings. **D**. Left: confusion matrix showing classification performance for sound (80 dB) versus no sound condition for an example recording. Right: decoding accuracy for classification of 80 dB sound events and no sound condition, comparing observed versus shuffled data across recordings. Black P value corresponds to Wilcoxon signed-rank test against chance value. Gray P value corresponds to Mann-Whitney U test with respect to shuffled labels. **E**. As in **D**, for classification of 60 dB sound events and no sound condition. **F**. As in **D**, for classification of visual stimulus versus no stimulus condition. **G**. Average z-scored motion energy weights for the first PC for the different trial types. P value from Kruskal-Wallis test. Individual datapoints represent individual recordings. N = 9 recordings, 7 animals.

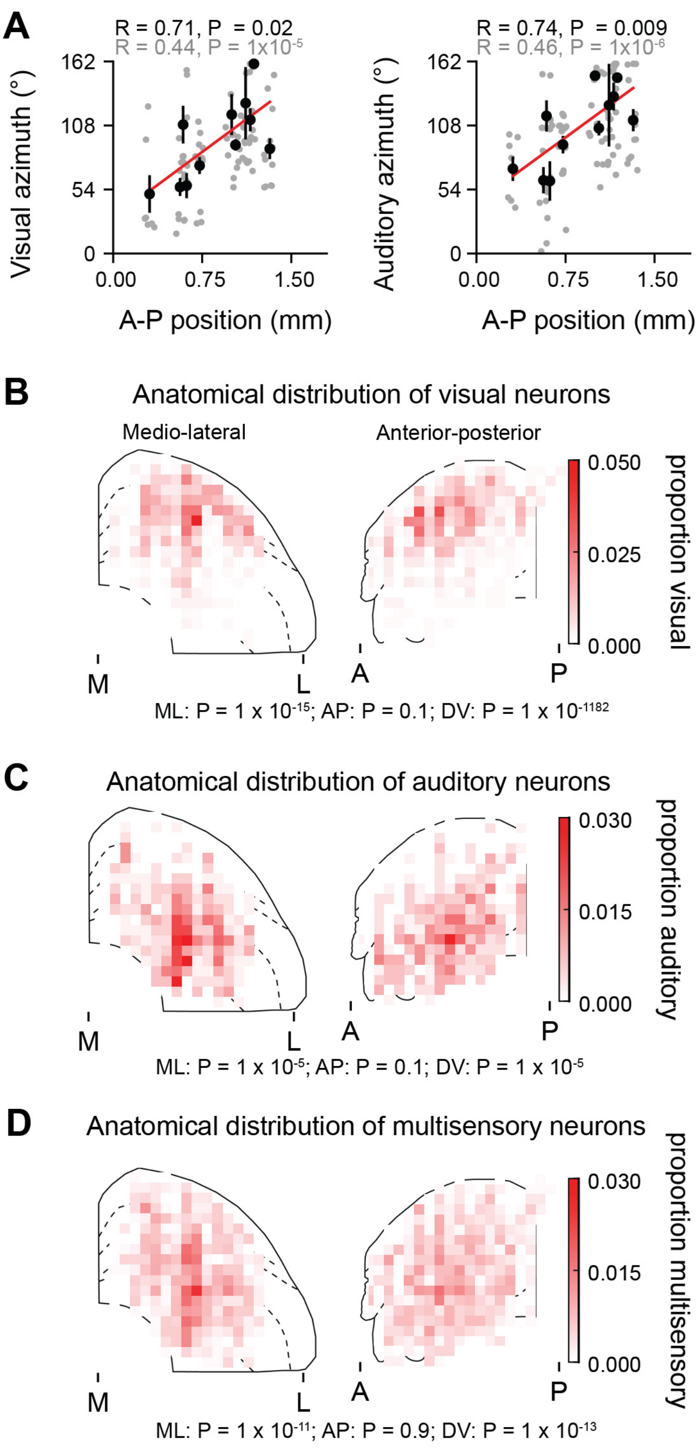

Supplementary Figure 2. Spatial correlates of receptive field properties and neuronal distribution in the superior colliculus

A. Correlation between RF azimuth and anatomical location along the AP axis for visual (left) and auditory (right) modalities. Data points represent individual neurons (gray circles) and individual recordings means (black circles). Error bars indicate the standard error of the mean across neurons within each recording. Red lines show linear regression fits. Visual map slope = 74 $\pm$ 25° /mm and offset = 30 $\pm$ 23°, auditory map slope = 73 $\pm$ 22 ° /mm, offset = 43 $\pm$ 20° offset. **B**. Anatomical distribution of visual neurons along the ML and AP axis. Left: ML axis (bins size: 102 µm ML x 120 µm DV). Right: AP axis (bins size: 89 µm AP x 120 µm DV). P values were calculated using a linear mixed model with mouse ID as random effect. **C**. Same as **B**, but showing distribution of auditory neurons. **D**. Same as **B**, showing distribution of multisensory neurons.

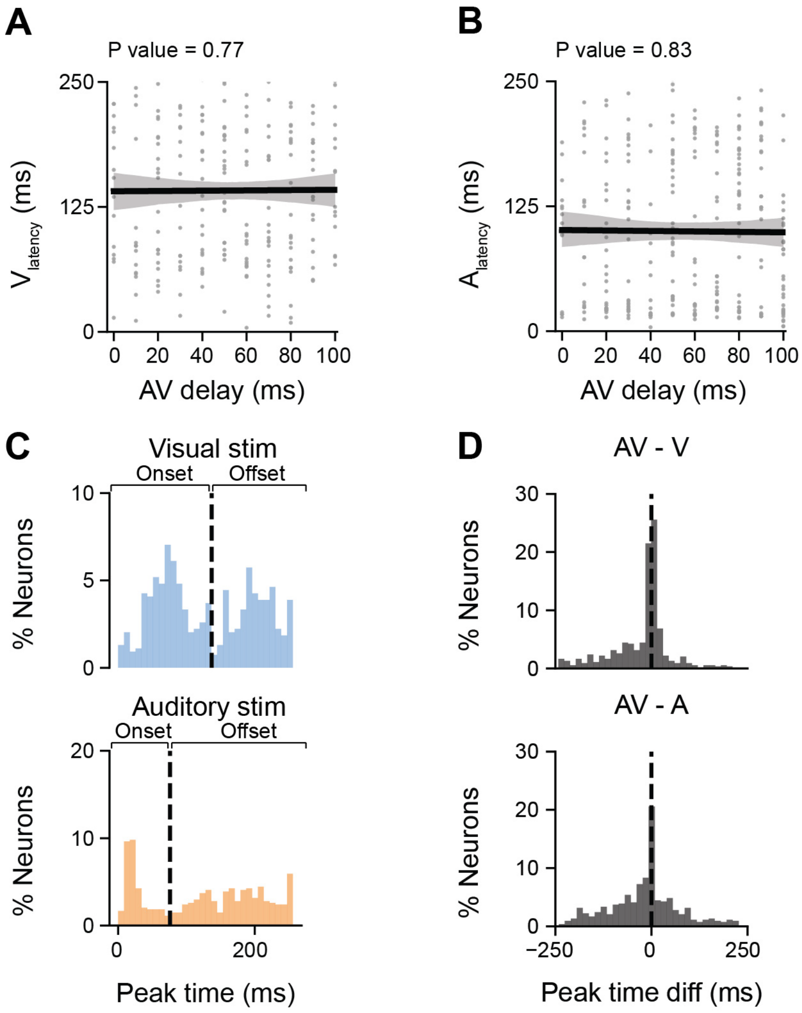

Supplementary Figure 3. Audiovisual delay is not explained by response latency to unisensory stimulation.

A. Preferred AV delay as a function of response latency to visual stimulation. Only delay-selective neurons that were responsive during visual stimulation were included (N = 232 neurons). P value from LMM. **B**. Same as **A**, for auditory response latency. Only delay-selective neurons that were responsive during auditory stimulation were included (N = 293 neurons) **C**. Distribution of response latency to visual (blue) and auditory (orange) stimuli for bimodal neurons. **D**. Distribution of differences in peak response latency for bimodal neurons. Top: difference between AV and visual peak response latency; Bottom: difference between AV and auditory peak response latency. N = 92 recordings, 24 animals.

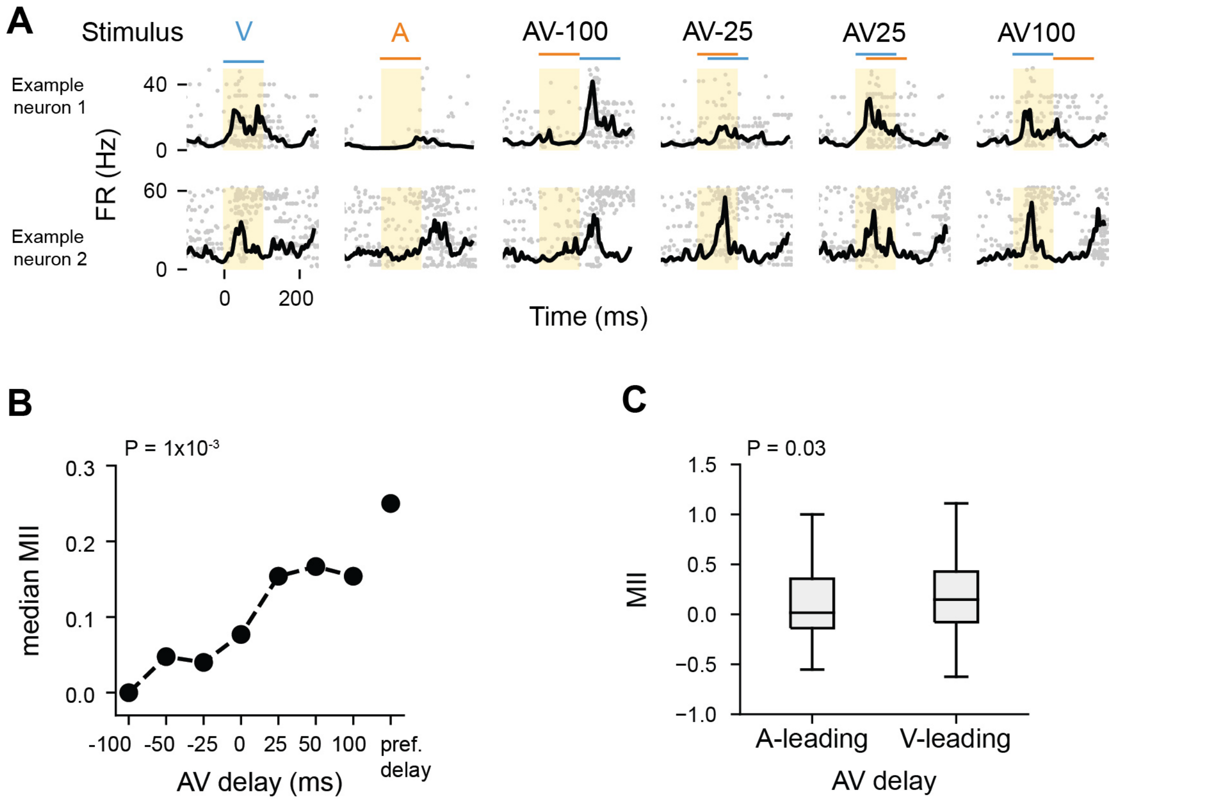

Supplementary Figure 4. Multisensory interactions are enhanced when visual stimulus leads on auditory.

A. Raster plots and PSTHs for two example neurons to unisensory and multisensory stimuli presented at variable delays. **B**. Quantification of median MII for each AV delay presented, as well as for the preferred delay across all delay neurons. P value from LMM. **C**. MII during auditory-leading stimuli and visual-leading stimuli. P value from LMM. N = 22 recordings, 6 animals.

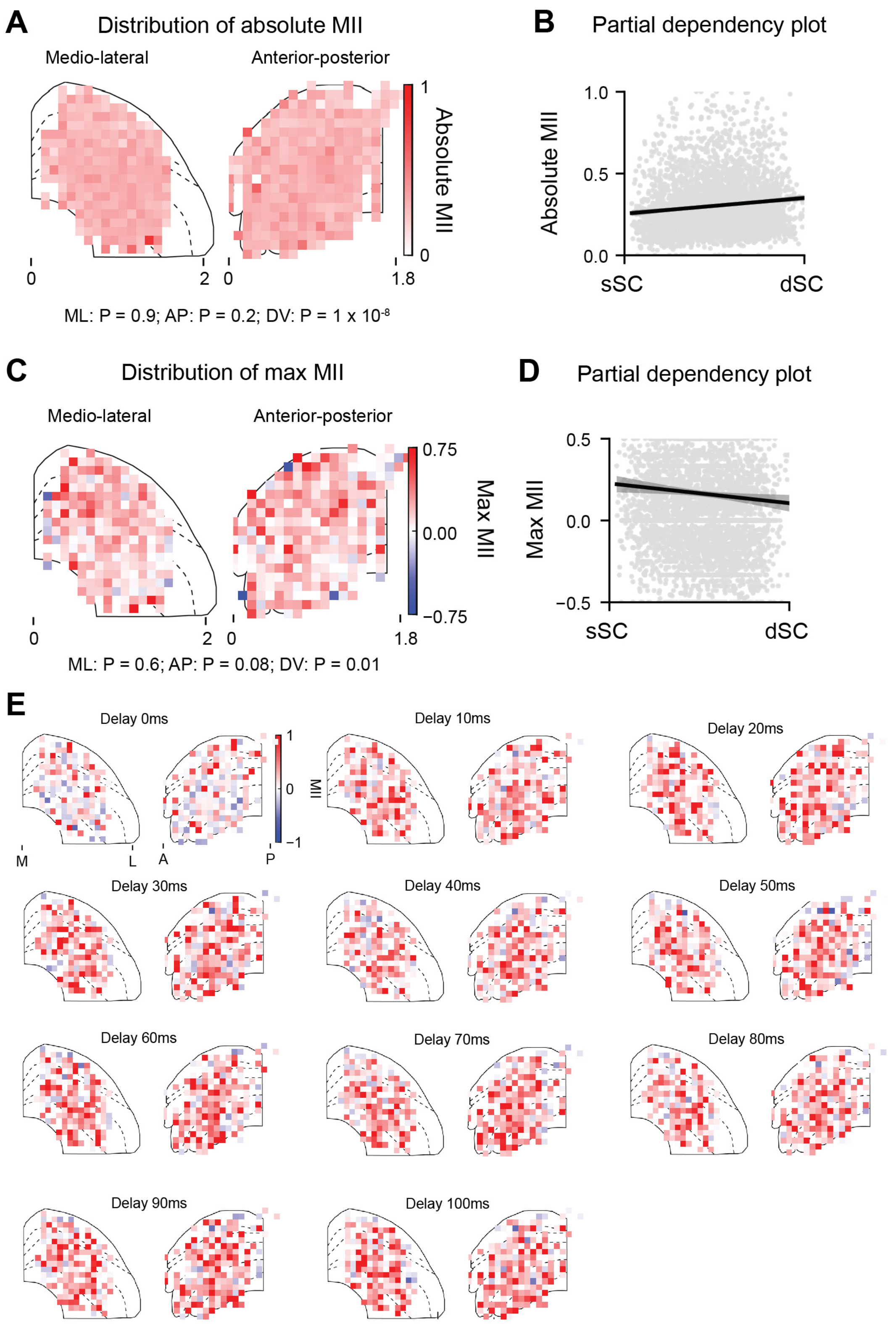

Supplementary Figure 5. Spatial distribution of neurons' multisensory index.

A. Mean absolute MII as a function of anatomical location. Left: ML axis (bins size: 102 µm ML x 120 µm DV). Right: AP axis (bins size: 89 µm AP x 120 µm DV). **B**. Partial dependency plot illustrating the linear gradient along the DV axis. P values were calculated using a linear mixed model with mouse ID as random effect. **C.** Same as **A** but for the maximum MII as a function of anatomical location. **D**. Same as **B** but for the maximum MII. **E**. MII distribution as a function of anatomical location for each AV delay. N = 92 recordings, 24 animals.

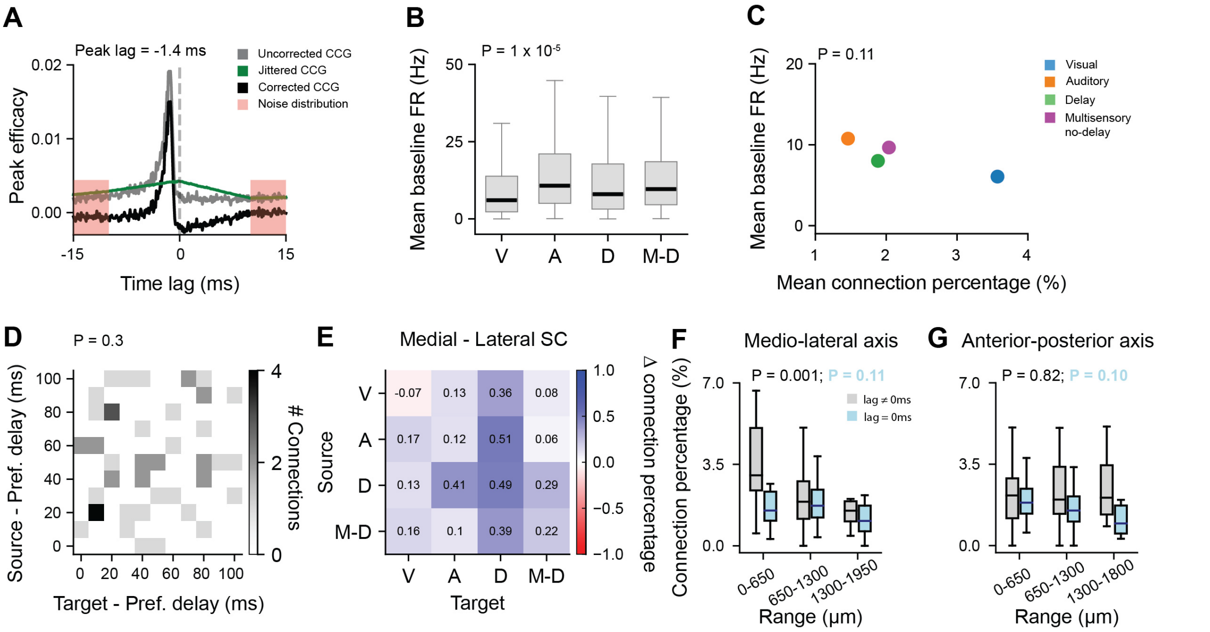

Supplementary Figure 6. Baseline firing rate of different functional subpopulations does not explain connection probabilities.

A. Jittered CCG based on all possible jitters within a 10-ms window and a noise distribution, which was estimated from the CCG flanks (10 to 15 ms before and after zero). The jittered CCG was subtracted from the original CCG to correct for slow timescale correlations larger than the jitter window[1,2]. A jitter-corrected CCG was considered significant if its peak occurred within 5 ms of zero and was more than five standard deviations above the mean of the noise distribution. **B**. Distribution of mean baseline FR for different functional subpopulations: V: visual, A: auditory, D: delay-selective neurons, M-D: multisensory delay-nonselective neurons. P value from LMM. **C**. Relationship between mean connection percentage and mean baseline FR for different functional subpopulations. P value from linear regression. **D**. Number of connections between source neuron and target neurons, binned according to their preferred delay. **E.** Matrix showing the difference in connection percentage between medial and lateral SC (bin size = 1.02 mm), for each neuronal subpopulation. Positive values (blue) indicate larger connectivity within a given class in the medial SC, whereas negative values (red) indicate larger connectivity in the lateral SC. N = 92 recordings, 24 animals. **F**. Connection percentage as a function of anatomical location along the ML axis of the SC for three equally spaced spatial bins. Connection percentage is displayed for both putative connections (1ms≤ lags ≤5ms; n connections: 2842) and for shared inputs (-1ms < lags <1ms; n connections: 2074). P value from Kruskal-Wallis. **G**. Same as **F** but for connections along the anterior-posterior axis.

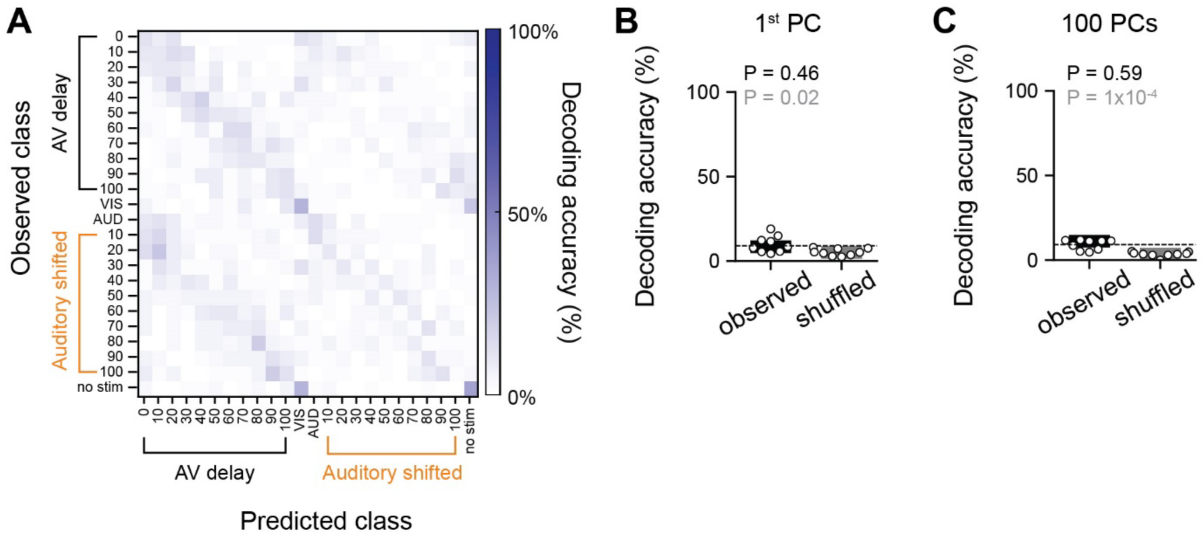

Supplementary Figure 7. Decoding audio-visual delays from motion energy representations.

A. Confusion matrix for AV delays discrimination using a decoder trained with the first PC for an example recording. **B**. Decoding accuracy for discrimination between AV delay conditions using a decoder trained with the first PC. Individual datapoints represent individual recordings. Black P value corresponds to Wilcoxon signed-rank test against chance value. Gray P value corresponds to Mann-Whitney U test with respect to shuffled labels. N = 9 recordings, 7 animals. **C**. As in **B**, for a decoder trained on the first 100 principal components from the mouse videos.

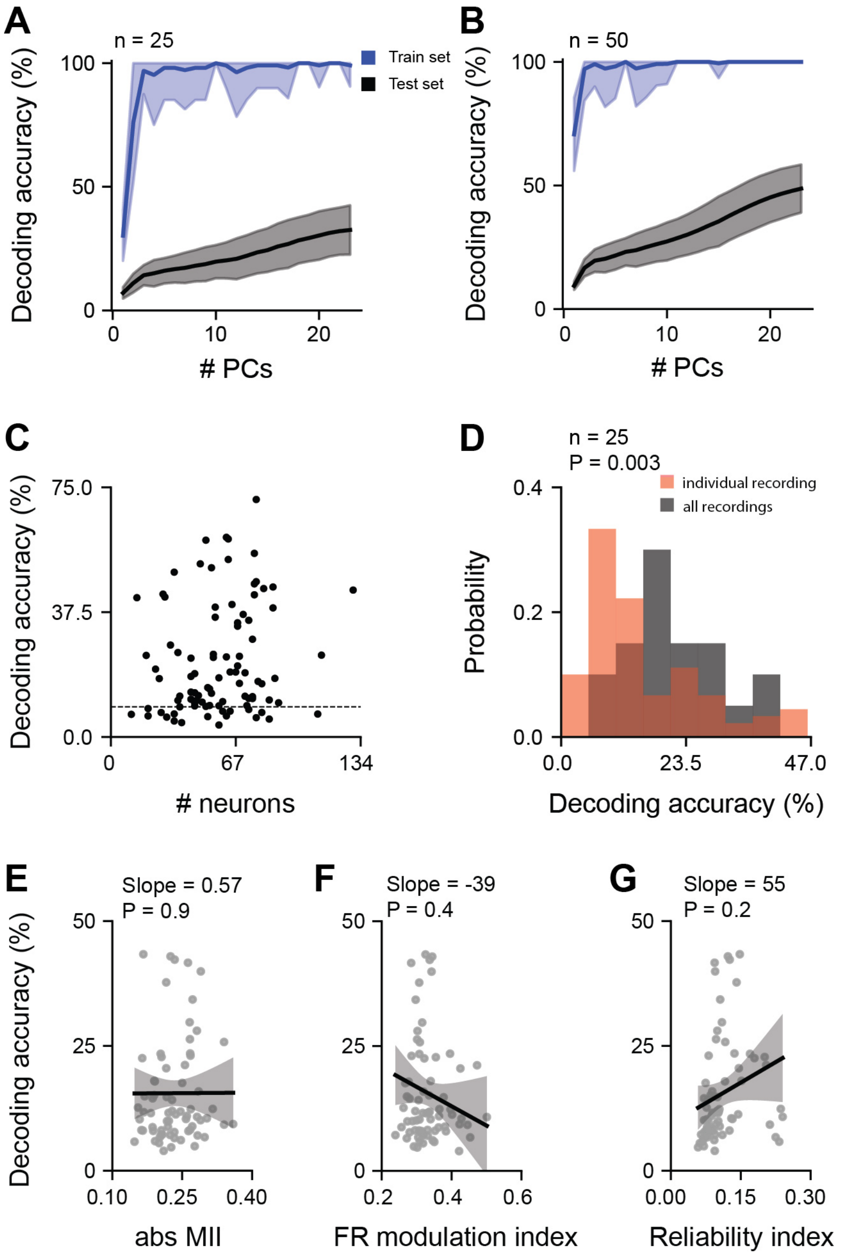

Supplementary Figure 8. The relationship between audiovisual delay decoding accuracy from individual recordings and single neuron response properties.

A. Decoding accuracy as a function of the number of principal components (# PCs) used in the SVM classifier reported for both the training set (blue) and the test set (black). Population size = 25 neurons. The shaded area represents the standard deviation over 20 repetitions. **B**. As **A** but for 50 neurons. **C**. Mean decoding accuracy as a function of the number of neurons for each recording. Each circle represents an individual recording (N = 92). The dotted line represents chance level. **D**. AV delay decoding accuracy obtained from simultaneously recorded neurons (red) and randomly selected neurons from different recordings (black). Number of neurons was fixed at 25. P value from Mann-Whitney U test. **E**. Mean classifier decoding accuracy for 25 neurons as a function of absolute MII. The black line represents the fitted regression line, while the shaded gray area denotes the 95% confidence interval. Gray circles correspond to individual recordings. P value from linear mixed model. **F**. as in **E**, for the FR modulation index. **G**. Same as **E**, for the reliability index. N = 92 recordings, 24 animals. An 80% training and 20% testing split with 5-fold cross-validation was applied across all SVM implementations. The analysis of decoding accuracy in panels **C-G** corresponds to the test sets.

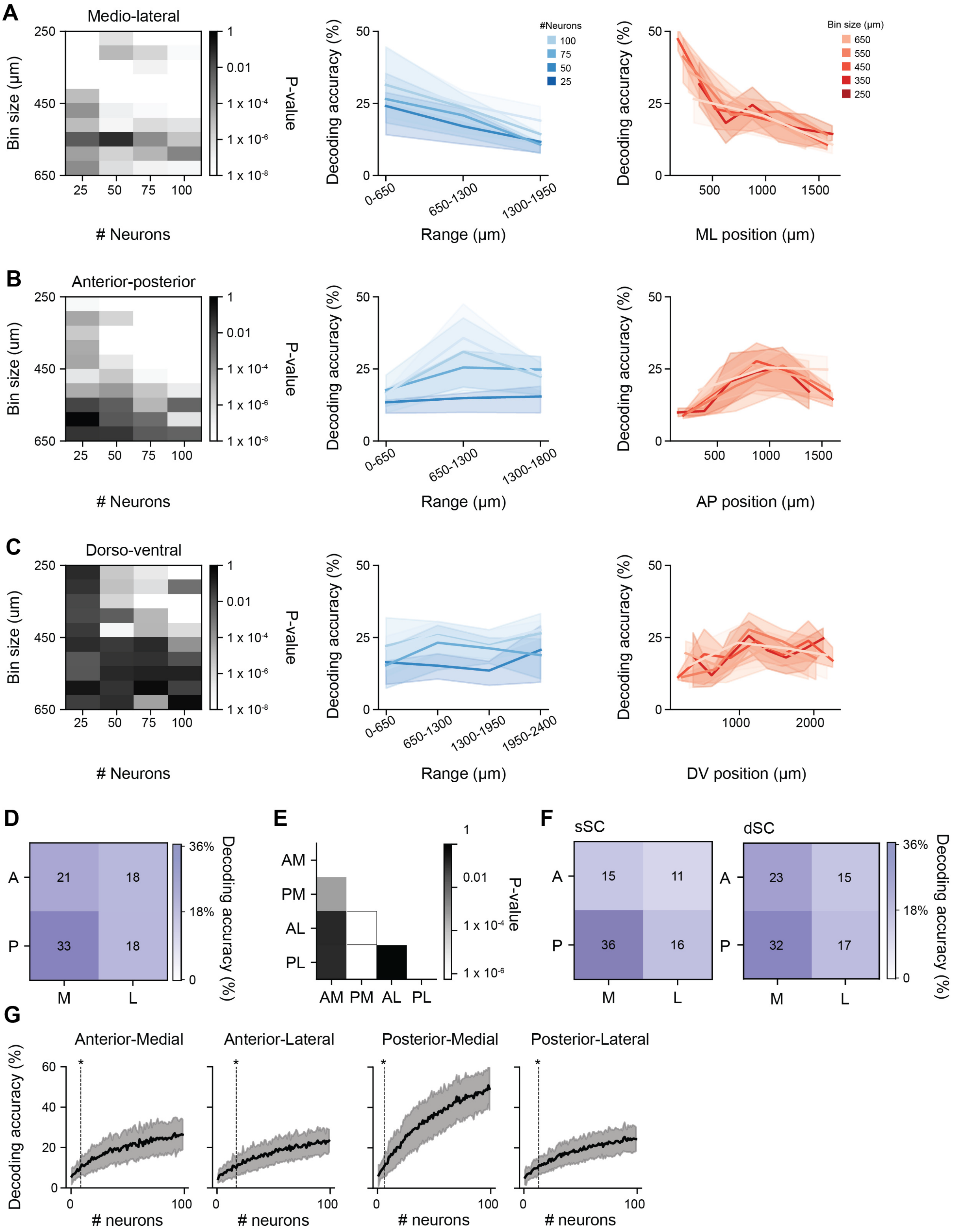

Supplementary Figure 9. Audiovisual delay decoding accuracy varies along the different anatomical axes of the SC.

A. AV delay decoding accuracy along the ML axis. Left: P values from Kruskal Wallis test on the mean AV delay decoding accuracy for different bin sizes and number of neurons included in the decoder. Centre: mean AV delay decoding accuracy for 25,50,75 and 100 neurons sampled from bins spaced by 650 µm along the ML axis. Right: mean AV delay decoding accuracy for 50 neurons for different bin sizes (from 250 to 650 µm). **B**. As in **A**, for the AP axis. **C**. As in **A**, for the DV axis. **D**. Mean decoding

accuracy for 50 randomly sampled neurons across 4 different subregions of the SC. Each bin covered 2.20 mm^3, with 1039 ± 121 neurons per bin. **E**. Dunn's post-hoc test corrected P-values for differences in classifier accuracy among the 4 subregions shown in panel **D**. AM: anterior-medial; PM: posterior-medial; AL: anterior-lateral; PL: posterior-lateral. **F**. As in D, for 50 randomly sampled neurons across 8 different anatomical subregions of the SC. Each bin covered 1.09 mm^3, with on average 528 ± 243 neurons per bin. Left: superficial SC (sSC); Right: deep SC (dSC). N = 92 recordings, 24 animals. **G**. Mean decoding accuracy as a function of number of neurons included in the classifier (black) for different anatomical bins. The shaded area represents the standard deviation over 100 repetitions. The dotted line represents the number of neurons necessary to achieve higher than chance performance. Anterior-medial, N = 9; anterior-lateral, N = 17; posterior-medial, N = 6; posterior-lateral, N = 13. An 80% training and 20% testing split with 5-fold cross-validation was applied across all SVM implementations. The analysis of decoding accuracy corresponds to the test set.

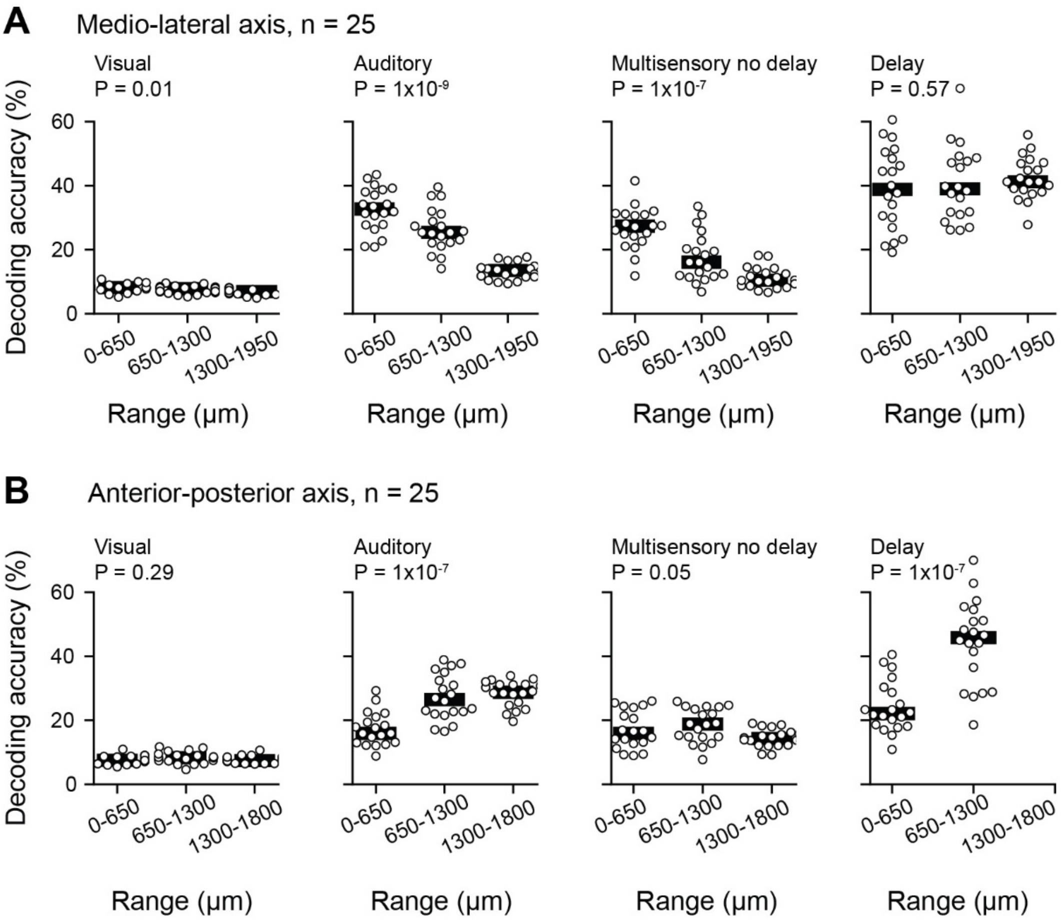

Supplementary Figure 10. Contribution of functional subpopulation to gradient of AV delay encoding in the SC.

A. AV delay decoding accuracy for different functional subpopulation as a function of ML axes. Each decoder was trained on the activity of 25 randomly sampled neurons, and the procedure was repeated 20 times. **B.** As in **A** as a function of AP axes. N = 92 recordings, 24 animals. An 80% training and 20% testing split with 5-fold cross-validation was applied across all SVM implementations. The analysis of decoding accuracy corresponds to the test set.

References

1. Harrison, M. T. & Geman, S. A Rate and History-Preserving Resampling Algorithm for Neural Spike Trains. *Neural Comput* 21, 1244–1258 (2009).

2. Smith, M. A. & Kohn, A. Spatial and Temporal Scales of Neuronal Correlation in Primary Visual Cortex. *Journal of Neuroscience* 28, 12591–12603 (2008).
