# Supplementary material for: Functional specialisation of multisensory temporal integration in the mouse superior colliculus
Source: Nat Commun. 2025 Oct 30;16:9615. doi: 10.1038/s41467-025-64600-x (PMC12575753; doi:10.1038/s41467-025-64600-x)
Supplement: Supplementary file 2 — Reporting Summary [file 41467_2025_64600_MOESM2_ESM.pdf]

nature portfolio

Corresponding author(s): Iacaruso, Florencia

Last updated by author(s): 2025/06/16

Reporting Summary

Nature Portfolio wishes to improve the reproducibility of the work that we publish. This form provides structure for consistency and transparency in reporting. For further information on Nature Portfolio policies, see our Editorial Policies and the Editorial Policy Checklist.

Statistics

For all statistical analyses, confirm that the following items are present in the figure legend, table legend, main text, or Methods section.

n/a	Confirmed	
☐	☒	The exact sample size (*n*) for each experimental group/condition, given as a discrete number and unit of measurement
☐	☒	A statement on whether measurements were taken from distinct samples or whether the same sample was measured repeatedly
☐	☒	The statistical test(s) used AND whether they are one- or two-sided<br>*Only common tests should be described solely by name; describe more complex techniques in the Methods section.*
☐	☒	A description of all covariates tested
☐	☒	A description of any assumptions or corrections, such as tests of normality and adjustment for multiple comparisons
☐	☒	A full description of the statistical parameters including central tendency (e.g. means) or other basic estimates (e.g. regression coefficient) AND variation (e.g. standard deviation) or associated estimates of uncertainty (e.g. confidence intervals)
☐	☒	For null hypothesis testing, the test statistic (e.g. *F*, *t*, *r*) with confidence intervals, effect sizes, degrees of freedom and *P* value noted<br>*Give P values as exact values whenever suitable.*
☒	☐	For Bayesian analysis, information on the choice of priors and Markov chain Monte Carlo settings
☒	☐	For hierarchical and complex designs, identification of the appropriate level for tests and full reporting of outcomes
☐	☒	Estimates of effect sizes (e.g. Cohen's *d*, Pearson's *r*), indicating how they were calculated

Our web collection on statistics for biologists contains articles on many of the points above.

Software and code

Policy information about availability of computer code

Data collection	OpenEphys (https://open-ephys.org/)<br>Bonsai (https://bonsai-rx.org/)<br>Matlab 2021a<br>Psychophysics Toolbox (http://psychtoolbox.org/)<br>ScanImage (Vidrio Technologies, https://www.mbfbioscience.com/products/scanimage/)<br>Stimulus apparatus (https://github.com/Iacaruso-lab/Coliseum)
Data analysis	MATLAB 2022b<br>Python<br>Facemap (https://github.com/MouseLand/facemap)<br>Kilosort2 (https://github.com/MouseLand/Kilosort/releases/tag/v2.0)<br>Phy (https://github.com/cortex-lab/phy).<br>Breinreg (https://github.com/brainglobe/brainreg)<br>ZETA test https://github.com/JorritMontijn/zetatest<br>Spikes (https://github.com/cortex-lab/spikes)<br>Matlab and python code for analysing the data and generating the figures is available at https://github.com/Iacaruso-lab/Bianchini2025_SC.

For manuscripts utilizing custom algorithms or software that are central to the research but not yet described in published literature, software must be made available to editors and reviewers. We strongly encourage code deposition in a community repository (e.g. GitHub). See the Nature Portfolio guidelines for submitting code & software for further information.

Data

Policy information about availability of data

All manuscripts must include a data availability statement. This statement should provide the following information, where applicable:

- Accession codes, unique identifiers, or web links for publicly available datasets
- A description of any restrictions on data availability
- For clinical datasets or third party data, please ensure that the statement adheres to our policy

Pre-processed data (spike sorted data) have been deposited in Figshare under accession code https://doi.org/10.25418/crick.28685360.v1 . Raw electrophysiological data has not been deposited due to size and similarity with pre-processed data but are available from the authors upon request. The open-source designs of the speakers and LED device and its associated circuits is available through our institute scientific hardware platform (https://github.com/Iacaruso-lab/Coliseum).

Research involving human participants, their data, or biological material

Policy information about studies with human participants or human data. See also policy information about sex, gender (identity/presentation), and sexual orientation and race, ethnicity and racism.

Reporting on sex and gender	N.A.
Reporting on race, ethnicity, or other socially relevant groupings	N.A.
Population characteristics	N.A.
Recruitment	N.A.
Ethics oversight	N.A.

Note that full information on the approval of the study protocol must also be provided in the manuscript.

Field-specific reporting

Please select the one below that is the best fit for your research. If you are not sure, read the appropriate sections before making your selection.

☒ Life sciences ☐ Behavioural & social sciences ☐ Ecological, evolutionary & environmental sciences

For a reference copy of the document with all sections, see nature.com/documents/nr-reporting-summary-flat.pdf

Life sciences study design

All studies must disclose on these points even when the disclosure is negative.

Sample size	Sample size was estimated based on the expected effect size based on similar studies (Hoy, et al., Current Biology, 2019, Ito, et al., PLoS Comput Biol 2021; Bimbard, C. et al. Nature Neuroscience 2023) and the current standard in mouse neuroscience studies.
Data exclusions	Recordings were spike sorted prior to data analysis. Units were only included in further analysis based on the criteria specified in the Methods. Recordings were excluded from further analysis if, upon visual inspection of the spike-sorted data and anatomical reconstruction of the probe track, the anatomical boundaries of the SC could not be determined. Data exclusion was done prior to analysis, in order not to bias exclusion criteria. Inclusion criteria for specific analysis are specified in the Methods.
Replication	Neuropixels experiments were performed in 24 animals (92 recordings in total). Each animal underwent a maximum of 5 probe insertions, with no more than two insertions per day. Statistics were performed across recordings. Mouse effect was used as a random factor effect when appropriate as specified in the Methods. Uninstructed movements were assessed in 7 mice (9 recordings in total) and each animal underwent a maximum of 2 recordings. Statistics were performed across recordings.
Randomization	The order of the anatomical location of the insertion was randomised. In all experiments the stimulus sequence was randomised in a block design, as described in the methods.
Blinding	Data analysis was performed blind to the anatomical location of the recording sites. All data collected went through the same data collection and analysis pipeline.

Reporting for specific materials, systems and methods

We require information from authors about some types of materials, experimental systems and methods used in many studies. Here, indicate whether each material, system or method listed is relevant to your study. If you are not sure if a list item applies to your research, read the appropriate section before selecting a response.

Materials & experimental systems

n/a	Involved in the study
☒	☐ Antibodies
☒	☐ Eukaryotic cell lines
☒	☐ Palaeontology and archaeology
☐	☒ Animals and other organisms
☒	☐ Clinical data
☒	☐ Dual use research of concern
☒	☐ Plants

Methods

n/a	Involved in the study
☒	☐ ChIP-seq
☒	☐ Flow cytometry
☒	☐ MRI-based neuroimaging

Animals and other research organisms

Policy information about studies involving animals; ARRIVE guidelines recommended for reporting animal research, and Sex and Gender in Research

Laboratory animals	Experiments in this study were performed in 31 male and female C57BL/6J.Cdh23753A>G mice (MRC Harwell, UK) aged 2–4 months.
Wild animals	This study did not involve wild animals
Reporting on sex	Data was collected from 4 females and 3 males for behavioural experiments and 13 females and 11 males for Neuropixels recordings. Sex-based analysis were not performed due to otherwise too small sample size.
Field-collected samples	The study did not involve samples collected from the field.
Ethics oversight	All animal procedures performed in this study were licensed by the UK Home Office and approved by the Crick Institutional Animal Welfare Ethical Review Panel (PEB4A5081 & PP2817210).

Note that full information on the approval of the study protocol must also be provided in the manuscript.

Plants

Seed stocks	N.A.
Novel plant genotypes	N.A.
Authentication	N.A.
